# Predicting the formation of NADES using a transformer-based model

**DOI:** 10.1038/s41598-022-27106-w

**Published:** 2024-02-22

**Authors:** Lucas B. Ayres, Federico J. V. Gomez, Maria Fernanda Silva, Jeb R. Linton, Carlos D. Garcia

**Affiliations:** 1 Department of Chemistry, Clemson University, 211 S. Palmetto Blvd, Clemson, SC 29634 USA; 2 Facultad de Ciencias Agrarias, Instituto de Biología Agrícola de Mendoza (IBAM-CONICET), Universidad Nacional de Cuyo, Mendoza, Argentina; 3 IBM Cloud, Armonk, NY 10504 USA

**Keywords:** Drug discovery, Chemistry, Analytical chemistry, Green chemistry

## Abstract

The application of natural deep eutectic solvents (NADES) in the pharmaceutical, agricultural, and food industries represents one of the fastest growing fields of green chemistry, as these mixtures can potentially replace traditional organic solvents. These advances are, however, limited by the development of new NADES which is today, almost exclusively empirically driven and often derivative from known mixtures. To overcome this limitation, we propose the use of a transformer-based machine learning approach. Here, the transformer-based neural network model was first pre-trained to recognize chemical patterns from SMILES representations (unlabeled general chemical data) and then fine-tuned to recognize the patterns in strings that lead to the formation of either stable NADES or simple mixtures of compounds not leading to the formation of stable NADES (binary classification). Because this strategy was adapted from language learning, it allows the use of relatively small datasets and relatively low computational resources. The resulting algorithm is capable of predicting the formation of multiple new stable eutectic mixtures (n = 337) from a general database of natural compounds. More importantly, the system is also able to predict the components and molar ratios needed to render NADES with new molecules (not present in the training database), an aspect that was validated using previously reported NADES as well as by developing multiple novel solvents containing ibuprofen. We believe this strategy has the potential to transform the screening process for NADES as well as the pharmaceutical industry, streamlining the use of bioactive compounds as functional components of liquid formulations, rather than simple solutes.

## Introduction

Over the past two decades, eutectic mixtures such as ionic liquids (IL)^1^, deep eutectic solvents (DES)^2^, and NADES (DES formed using natural compounds)^3^ have been extensively explored as green alternative liquid media to traditional organic solvents^4–9^, and are applicable in a wide variety of industries targeting sustainable chemistry^10–12^. These new materials are composed of specific ratios of two or three components, often in solid state, that lead to a material featuring a melting point that is significantly lower than the melting points of the individual components; often below room temperature^13^. Among those, mixtures leading to the formation of liquids that are stable at room temperature are the most important. A careful selection of the physico-chemical characteristics of the components (molecular weight, hydrogen bonding, pKa, etc.) enables the formation of solvents with different properties (e.g., stability, viscosity, polarity, pH, conductivity, permittivity, and density)^14^. This aspect is of great interest in tailoring solvents towards several applications including biocatalysis^15–18^, chromatography^19–22^, extraction media^23–28^, electrochemistry^29–31^, as well as pharmaceutical ingredients to enhance availability and/or therapeutic properties^32–35^. It is also important to note that among these solvents, DES and NADES are considered more environmentally friendly than IL due to their intrinsic properties such as biodegradability^36^, low or non-toxicity^37^, easy preparation with no purification steps^38^, and inexpensive starting materials^39^.

While NADES represent one of the most promising and fastest-growing DES, their development is (until now) almost exclusively empirically driven. Because there exist only general guidelines to explain (but not predict) their formation, new NADES are often derived from structurally similar components considering general properties of the precursors such as hydrophobicity, number of functional groups, and number of donors or acceptors of hydrogen bonds. Examples of these include families of NADES based on choline chloride^40^, carbohydrates^41,42^, or organic acids^43^. A second drawback, broadly limiting the development of new NADES is that bench-top trials of new mixtures are time-consuming, labor-intensive, and expensive even at a laboratory scale. Aiming to provide insights into the relationship between chemical structure and properties of NADES/DES and guide the application of these mixtures, computational simulations^44,45^ have recently gained popularity. Generally speaking, these approaches vary from thermodynamic modeling such as Perturbed Chain—Statical Associating Fluid Theory (PC-SAFT)^46^ to atomistic modeling methods including Density Functional Theory (DFT) at the quantum level^47^. Although these models are certainly capable of explaining several properties of eutectic mixtures, they require specialized knowledge to build and are not yet able to make statistically-validated predictions of new mixtures. In parallel to the methods cited above, machine learning approaches based on artificial neural networks (ANN)^48^ can also be used as an auxiliary tool to predict physicochemical features of solvents^49–52^. However, due to the complexity of these deep learning architectures, a substantial volume of data would be required to create such a model *from scratch*, to properly train the parameters of the neural network (i.e., weights and biases), and to extract meaningful information from the chemical space. As of today, the development of a database containing enough chemical information seems to be an insurmountable task, at least from the experimental point of view.

In this context, we propose a much simpler solution to predict the mixtures of natural compounds that would lead to the formation of stable NADES. The approach is based on the use of a transformer-based^53^ neural network model by means of Simplified Molecular-Input Line-Entry System (SMILES)^54^ representations, rather than an extensive set of physicochemical parameters as input. This strategy—adapted from language learning—allows the use of relatively small datasets; which also reduces training time, model complexity, and computational cost. It is important to mention that similar transformer-based approaches have been successfully applied for other subfields of chemistry including prediction of organic synthesis^55^ and retrosynthesis^56,57^, conversion of chemical notation^58,59^, molecular geometry learning^60^, and prediction of regio- and stereoselective reactions^61^. However, to best of our knowledge, this is the first report describing the use of machine learning (and more specifically, transformers) towards the guided design and screening of new deep eutectic solvents. Briefly, the approach consists of pre-training a transformer model by using unlabeled general chemical data, and then fine-tuning the last layer of neurons in the model to perform a binary classification using labeled chemical data related to NADES. Our results demonstrated satisfactory performance (F1-score = 0.82), allowing the prediction of multiple stable eutectic mixtures (n = 337) from a general database. The validity of such predictions was verified by comparing the stability those NADES against those reported in previous publications as well as by the development of new solvents containing ibuprofen, a compound that despite being one of the most important over-the-counter analgesics, displays limited therapeutic potential due to its poor solubility in water.

## Results

**Figure 1:**
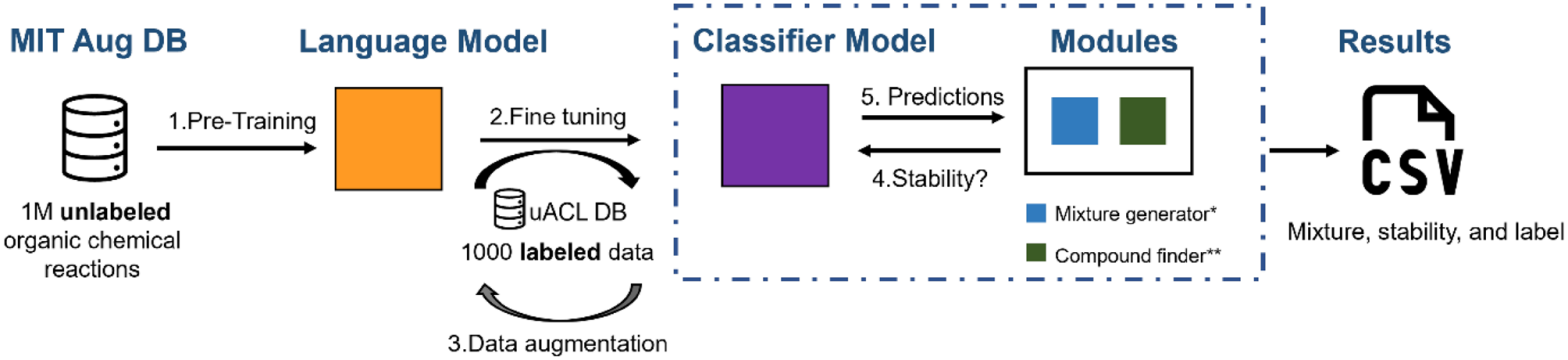
Rational design implemented to predict the stability of NADES.

Aiming to facilitate the discussion, we present a general overview of the rational design to predict the stability of eutectic mixtures. The first step consists in training a neural network model using general unlabeled chemical data from a large corpus of organic reactions using the “SMILES” text-based representation; the specific training task at this stage is the ELECTRA variant of Masked Language Modeling (MLM) (https://huggingface.co/docs/transformers/model_doc/electra#transformers.ElectraForMaskedLM). Next, the task of the Neural Net is changed from MLM by swapping the last layer of neurons for a freshly-initiated Binary Classifier layer; then the model is fine-tuned using the labeled NADES/DES dataset, also using the SMILES format but with the addition of special characters to represent the stoichiometric ratios (which are not present in the pre-training dataset). Additionally, an auxiliary program (uACL software) is used to infer the probability of any mixture to form a stable NADES/DES and then export the results (e.g., mixture composition, stoichiometric ratio, and probability of stability) in CSV tabular format. Figure 1 schematically shows the modules and the work sequence.

The performance metrics for evaluating each step described in Fig. 1 will be also discussed in this section; our primary goal is to use those metrics in conjunction with chemical knowledge to statistically support the development of the proposed strategy.

### Pre-training a general chemistry model from scratch

**Figure 2:**
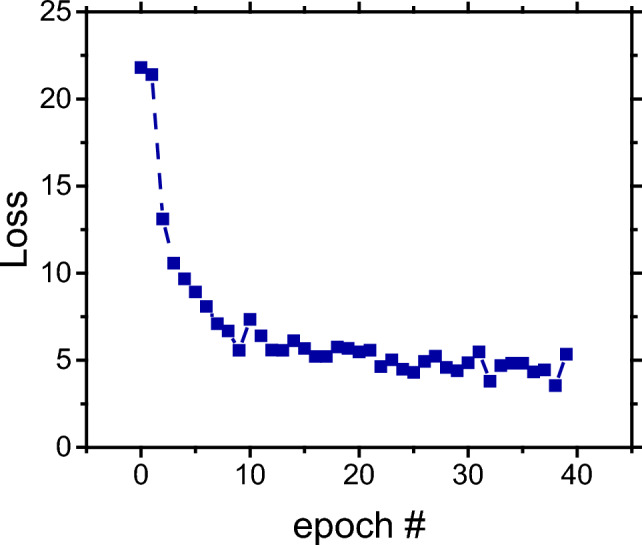
Dependence of the loss function as a function of epoch number obtained during training process for Masked Language Modeling (MLM) and using the MIT mixed augmented database.

In this first step, approximately one million unlabeled organic chemical reactions (Molecular Transformer MIT Mixed Augmented^55^) SMILES notation canonicalized by RDKit^62^ were used as text dataset to pre-train the transformer model. Typically, this process requires a large amount of unlabeled data since the neural network will try to model a language based on text sequences within specific contexts, continuously adjusting their intrinsic parameters (weights and biases) to minimize the output of the loss function (i.e. the “loss”)^63^. The loss versus epoch graph for the pre-training step is shown in Fig. 2.

Under the experimental conditions previously described, the training process was completed in approximately 12 h (18 min per epoch) using the MIT augmented database. As a point of reference, the same training process would take 21 days in a medium tier desktop computer equipped with a GTX 1050 Ti GPU.

It is important to note that the loss function dramatically decreases from epochs 0 to 10 and then remains relatively constant until the end of the training process. The average loss for the training dataset was 3.87 while the average loss for the test dataset was 4.00, suggesting that the performance of the neural network using both datasets reached a convergence point where more training is unlikely to lead to an improvement in the general chemistry model. At this point, the training process was stopped, and the generated model was tuned under several augmented data conditions.

### Fine-tuning a general chemistry model into a binary classifier for NADES and DES mixtures

The fine-tuning step was carried out by using the uACL DB developed and curated by our research group which contains approximately 1000 labeled mixtures of DES and NADES reported in the literature. While these mixtures may not represent the best ratios (leading to the absolute lowest possible melting point), they have all been classified as DES/NADES in peer-reviewed publications. Within these, 800 mixtures are stable (labeled as 1), and 200 mixtures are not stable (labeled as 0). While this reflects what is normally published (mostly positive results), the imbalance is likely to have undesirable effects on the model such as classification bias^64^, impacting the performance of the classifier (e.g., overoptimistic estimation). Aiming to overcome this issue, a data augmentation strategy was devised by generating random compound mixtures in the training dataset, which were labeled as zero (unstable). This approach is supported by the idea that the probability of generating a stable eutectic solvent by randomly mixing chemicals at random stoichiometric coefficients, is simply very low. For example, varying the stoichiometric coefficient between 1 and 10 for the 198 compounds in our database, leading to approximately 40 million possible ternary combinations $\left( {\frac{618!}{{3!{ } \times { }615!}}} \right)$. In this sense, the effectiveness of this strategy was mainly assessed by evaluating two parameters: Matthews correlation coefficient (MCC)^65^ and loss function. For both cases, the training dataset was augmented by adding synthetic data (1, 5, 10, 25, 50, 100, and 500 random mixtures) while the test dataset remained constant with 200 mixtures.

### Assessing the Matthews correlation coefficient

**Figure 3:**
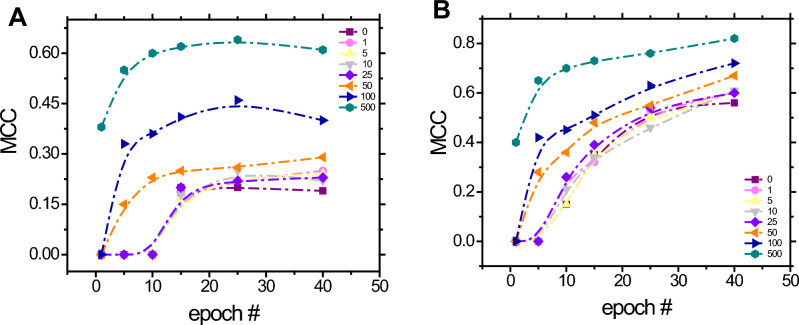
Effect of training data augmentation (1, 5, 10, 25, 50, 100, and 500) on MCC versus number of epochs number for test dataset (**A**) and training dataset (**B**). The MCC value represents the agreement between the predicted value and the true class, where: + 0.01 to + 0.19 indicates no or negligible relationship, + 0.20 to + 0.29 indicates weak positive relationship, + 0.30 to + 0.39 indicates moderate positive relationship, + 0.40 to + 0.69 indicates strong positive relationship, and + 0.70 or higher indicates very strong positive relationship.

The Matthews correlation coefficient has been successfully applied as a reliable metric for binary classification problems where the dataset available for training as well as fine tuning is unbalanced^66–68^, as in our application. This metric takes into consideration all of the categories in the confusion matrix^69^ (true positive, false negative, true negative, and false positive) to compute the correlation between the predicted value by the classifier with the true one. This correlation ranges from − 1 to + 1, where − 1 indicates total disagreement, 0 indicates no correlation, and + 1 indicates total agreement. A detailed explanation of the advantages of using MCC over traditional metrics for machine learning such as F1-score and accuracy can be found elsewhere^70^. The effect of several training data augmentation on the MCC metric versus number of epochs is shown in Fig. 3.

For the testing dataset (Fig. 3A), the MCC rises as the epoch number increases, reaching a plateau in the iteration number 15 for all augmented data scenarios. Additionally, it is interesting to note that the MCC is improved as the training synthetic data is incremented, accomplishing a satisfactory performance (MCC higher than 0.40) by using 100 and 500 augmented data after the iteration number 15. On other hand, the model’s performance evaluating the training dataset (Fig. 3B) is already satisfactory even without implementing any synthetic data (black line). This is expected since the same dataset was already seen by the deep neural network during the fine-tuning process. Additional information related to the confusion matrix used for calculating the test MCC at 15 epochs for both 100 and 500 synthetic data can be found in the supplementary information (Table SI 1 and Table SI 2).

### Assessing the loss Function

**Figure 4:**
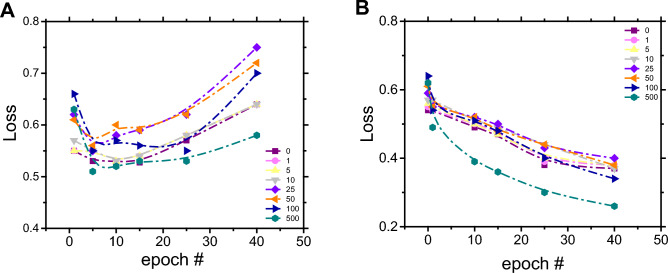
Dependence of the loss function as a function of epoch number obtained during the evaluation process for the binary classifier using the test dataset (**A**) or the training dataset (**B**). Series in each figure corresponds to the number of datapoints in the augmentation dataset.

The measurement of the loss function was performed aiming to elucidate the effect of the implemented data augmentation strategy on the classifier’s performance in terms of underfitting as well as overfitting. The results are shown in Fig. 4.

**Figure 5:**
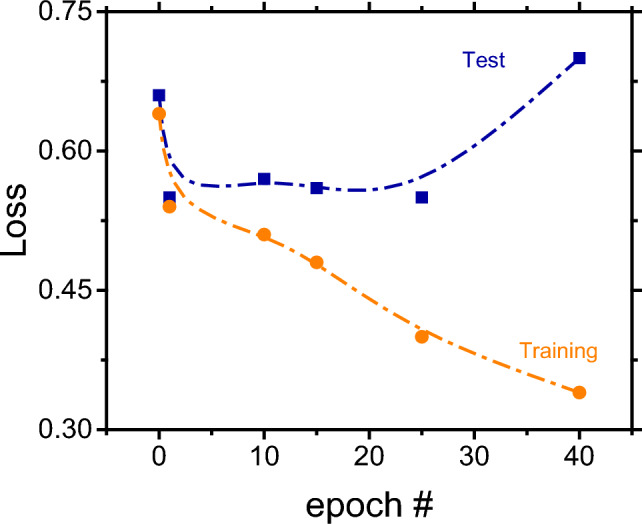
Dependence of the loss function as a function of epoch number obtained during the evaluation process for the binary classifier using the test dataset (blue square boxes) or the training dataset (orange circles), both considering 100 datapoints in the augmentation dataset. Line included to guide the eye.

The results presented above suggest that the classifier starts to overfit after iteration 15 regardless of the amount of data augmentation (Fig. 4A). As expected, this issue is not apparent upon assessment of the training dataset (Fig. 4B), where the loss decreases as the number of synthetic data is added. The overfitting problem is clearly evidenced when the loss for the test and training dataset is plotted together for the same number of synthetic data (100), as summarized in Fig. 5.

Initially, both losses were at same point (approximately 0.67) and then they start to diverge as the epoch number increases, reaching the maximum difference at epoch #40. At this point, the model was considered to be overfitted and thus provided a rather poor generalization of unseen data. In contrast, the classifier trained only with 5 epochs was underfitted. In this case, the training time was too short, and the model was not able to get meaningful information from the chemical space. In this context, an optimum classifier should be trained with the number of epochs in between these two extremes, where the number of interactions is enough to learn important information from the dataset but not enough to overfit by "memorizing” noise and other un-useful information in the training data.

### Predicting the stability of mixtures containing Ibuprofen

Ibuprofen [2-(4-isobutylphenyl)propionic acid] is a relatively safe^71^, well-known non-steroidal anti-inflammatory drug (NSAID) that is sold world-wide to treat mild to moderate pain, inflammation, and fever. Since its introduction in the market back in 1970s, ibuprofen has become one of the most commonly used NSAIDs^72^ and represents a global market of more than $7500 M per year. Its effects are due to the inhibitory actions on cyclo-oxygenases, which mediate the synthesis of prostaglandins^73^. Despite these advantages, ibuprofen is poorly soluble in water and therefore its bioavailability is limited by the dissolution of the solid form(s). Aiming to address this problem, various formulations of ibuprofen have been proposed including the use of prodrugs, inclusion complexes, microencapsulation and dispersion in various solvents^74–76^. Despite these advances, the solubility of ibuprofen is today a limiting factor that hinders the development and applicability of oral, injectable, and topical preparations of this drug^77,78^. Considering that a liquid form of ibuprofen could potentially improve the bioavailability of the drug while exhibiting fewer side-effects than some current formulations, we propose the use of our algorithm to develop a set of NADES based on ibuprofen. While as of today there are three reports describing the formation of ibuprofen-based DES/ILs in the literature^79–81^, it is important to note that those strategies are derivative from previously-reported DES and that none of those systems can be directly translated to other pharmaceuticals.

**Figure 6:**
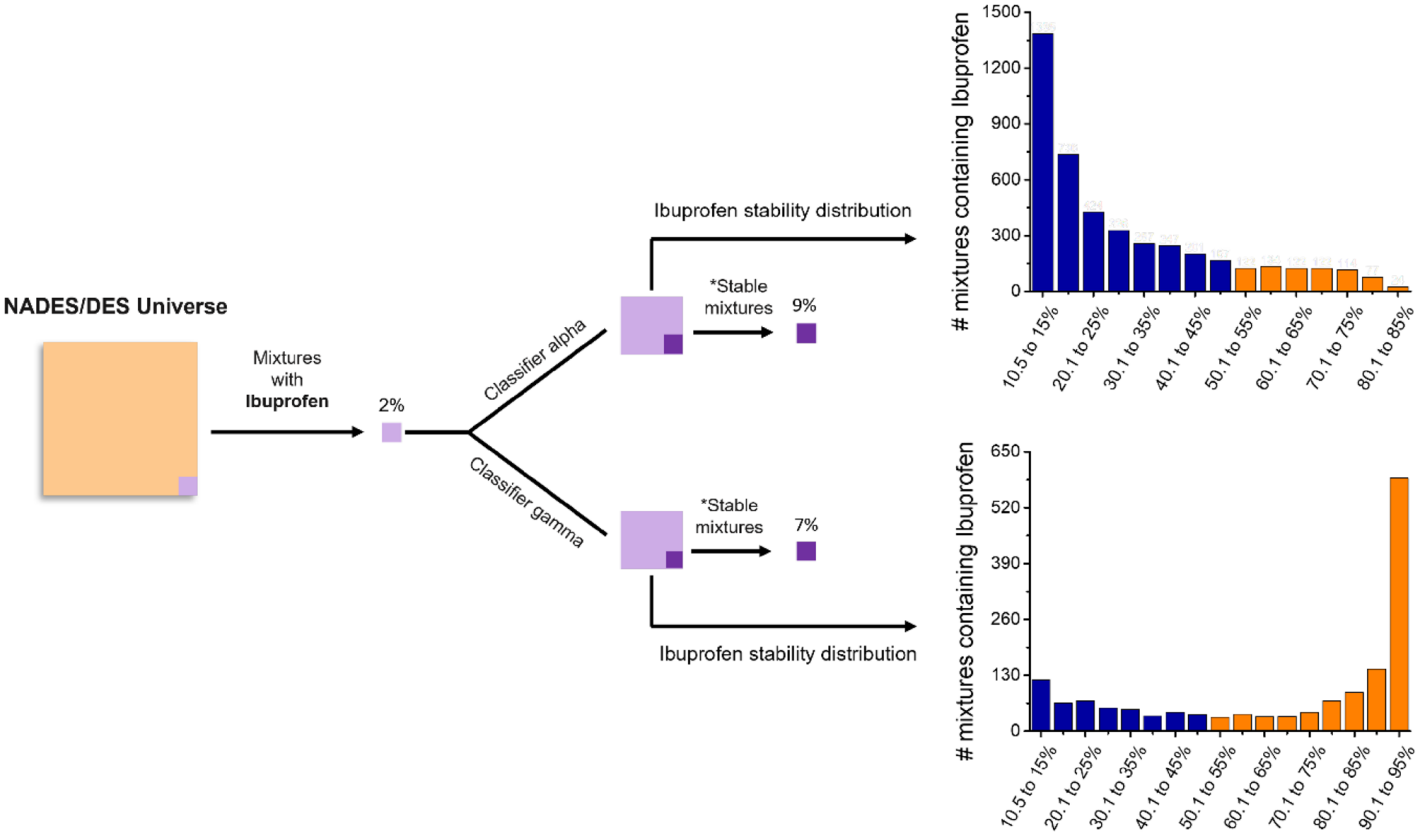
Stability score distribution of mixtures containing ibuprofen predicted by Classifier Alpha (top) and by Classifier Gamma (bottom). The mixtures included in the most likely group of the histogram (80.1–85%) obtained with classifier Alpha are further discussed and experimentally validated in this work.

Toward these ends and based on the results described in Sect. 3.2, a classifier (designated as classifier Alpha) was designed to represent an ideal model for our application. This classifier was fine-tuned by using the training data set augmented with 100 synthetic data and the number of epochs was fixed at 15. For comparison purposes, another classifier (designated as classifier Gamma) was also fine-tuned by using the same augmented dataset but with the number of epochs set to 40. Both classifiers were used to predict the stability of 1 million unlabeled candidate mixtures (collectively labeled the NADES/DES Universe) which were randomly generated by the uACL software. It is important to state that those mixtures present in the NADES/DES Universe were randomly generated, rather than fixing their constituents to a specific component such as ibuprofen or any other chemical. Posterior the predictions, only the results (compound mixture in SMILES format, predicted stability score, and label) for mixtures containing ibuprofen were post-processed and then exported in the CSV format. The distribution of stability scores for mixtures containing ibuprofen as predicted by both classifiers are shown in Fig. 6.

Using this strategy, approximately 2% out of the NADES/DES Universe presented ibuprofen in its composition. Within these, 9% of those mixtures were predicted to be stable by classifier Alpha; while this percentage dropped to 7% by using classifier Gamma. It is important to mention that we defined stable mixtures as the ones where the score of the “Stable” classification—a rough approximation of predicted probability—was higher than 50%. Therefore, the number of stable mixtures could be considerably smaller if this cut-off was set to 70%, for example. Additionally, the database used for generating those random mixtures is biased with compounds known to produce eutectic solvents (e.g., hydrogen bond donors and acceptors). As a point of reference, the same strategy was implemented by using an open-source database of natural compounds^82^ and the percent of mixtures predicted to be stable was less than 1%.

It is important to note that classifier Alpha predicts a decreasing stability distribution, presenting only 24 mixtures at range of predicted stability score between 80.1 and 85%. On the contrary, classifier Gamma has an increasing distribution for that probability, presenting more than 600 mixtures with the probability of forming a stable NADES between 90.1 and 95%. This difference was somewhat expected since this classifier was trained to be overfitted although it’s MCC has been the same as the classifier Alpha (0.42), indicating that the number of training iterations plays an important role during the development of an optimal classifier. Moreover, from a statistical point of view, it is more likely that the number of eutectic mixtures decreases as the probability of being stable increases as exhibited by the classifier Alpha. Out of those mixtures containing ibuprofen predicted by this classifier, we decided to further consider the 10 most likely to form stable NADES (highest probability of rendering a stability of 1). These mixtures and the respective probability to form a stable NADES are summarized in Table 1.

**Table 1:** Composition for the 10 mixtures (containing ibuprofen) most likely to form a stable NADES, as computed by classifier Alpha.

Mixture #	Component 1	Component 2	Component 3	Molar ratio	Probability (%)
1*	Diethanolamine	Ibuprofen	Glycol	1:2:1	84.5
2*	1,2-Butanediol	Ibuprofen	Methanol	1:1:3	83.8
3	Ibuprofen	Glycol	1,2-Butanediol	1:1:2	83.6
4*	Methanol	Undecanoic Acid	Ibuprofen	2:1:1	83.4
5	Proline	Choline Chloride	Ibuprofen	2:2:1	83.3
6	Undecanoic acid	Ibuprofen	Glycol	1:1:5	82.5
7*	Proline	Ibuprofen	Diethanolamine	1:3:3	82.3
8*	Sodium acetate	Methanol	Ibuprofen	1:2:1	82.0
9	Choline Chloride	Ibuprofen	Glycol	1:3:4	81.2
10	Mannitol	Choline Chloride	Ibuprofen	2:1:3	80.8

Mixtures containing methanol (neurotoxic) and diethanolamine (not natural) are included but marked with *, as these mixtures were only considered to demonstrate the applicability of the proposed approach.

The number of mixtures selected to demonstrate the applicability of the approach (10 most likely out of the 24 predicted using a threshold of > 80.1%) was selected as a balance between the number of cases and the resources needed to synthetize the NADES. All the solvents presented in Table 1 are ternary mixtures with well-known hydrogen bond acceptors as well as hydrogen bond donors on it is a composition such as chloride derivates^83^, alcohols^84^, acids^85^, and polyethylene glycol^86^. In contrast, most of the unstable solvents predicted by this classifier (Table SI 3) are quaternary and/or quinary mixtures with a high number of molar ratios. These trends were somewhat expected due to the chemical complexity of NADES formed by 10 molecules or more.

### Experimental validation of the predicted eutectic mixtures

In order to demonstrate the validity of the predictions provided by the proposed approach, the 10 combinations most likely to form stable NADES (Table 1) were prepared in the laboratory. In cases, the corresponding amount of the pure constituents were mixed in a sealed glass vial and incubated at 80 °C (in a water bath), under gentle stirring, for approximately two hours. This process rendered liquid mixtures that were then removed from the water bath and placed on the bench, where they were kept at room temperature for (at least) a week. It is also important to note that those mixtures formed with methanol (marked as * in Table 1) are not strictly considered natural and would not be applicable towards pharmaceutical preparations. However, those mixtures were still experimentally evaluated with the purpose of validating the predictions from the algorithm.

**Figure 7:**
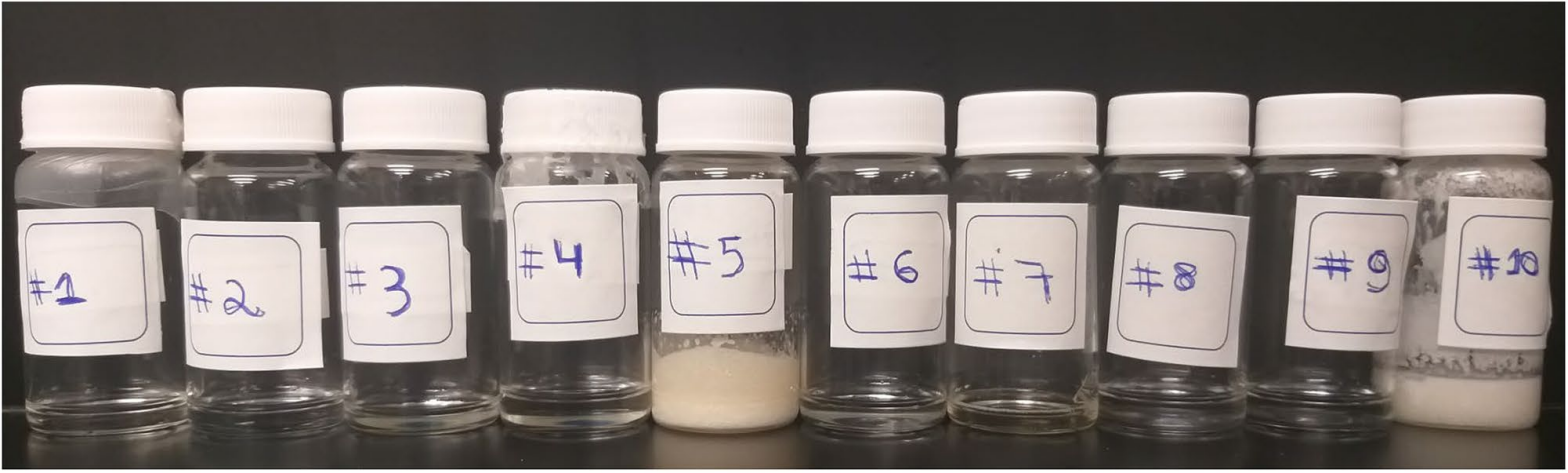
Experimental validation of the formation of the NADES predicted by classifier Alpha. The composition of each mixture is described in Table 1.

As shown in Fig. 7, eight out of the ten mixtures (80%) rendered stable NADES, remaining in clear liquid form at room temperature for at least one week. As this is very close to the average predicted stability score of these mixtures (82.8%), this provides some indication that the predicted stability scores may be a good proxy for the actual probability of forming a stable NADES. In ideal training conditions—neither under-nor overfitting and with a relatively unbiased training dataset—a classifier’s prediction scores should be a good approximation of true probability. This finding also suggests that while this paper only explored the top 10 mixtures, a more extensive list including all 24 mixtures at range of probability for a stable NADES between 80.1% and 85% could provide additional stable formulations.

Furthermore, when classifier Alpha’s performance was tested on the test dataset (see Sect. 3.5 below), its overall accuracy was calculated at 82%: a strong suggestion that the performance on the held-back test dataset corresponds very well to the model’s true predictive power. More extensive testing would be required to verify the model’s predictive power with certainty, both with regard to its overall accuracy (approximately 82% of its predictions are correct when a threshold of 0.5 is used) and its confidence in specific individual predictions of stability (averaging 82.8%) but these results are strongly suggestive of a good correspondence on both counts.

Although the melting points of many of the constituents are well above room temperature, the corresponding mixtures do not render crystals in the NADES; this is attributed to their participation in hydrogen bonding, leading to the eutectic mixture. For example, the melting point of sodium acetate (in mixture #8) is 324 °C so it would normally remain solid at room temperature. The stable liquid form of the NADES suggests that the combination of ibuprofen and methanol hinders the formation of crystals through this mechanism^87^. Moreover, while an equimolar mixture of ibuprofen and sodium acetate without methanol also rendered a stable NADES, this mixture also featured high viscosity. This observation supports the hypothesis that protic solvents such as methanol can be used to adjust the viscosity of NADES as well as DES^88–90^. Additional information about the physicochemical properties (e.g., number of hydrogen bond donor and acceptor) of ibuprofen, sodium acetate, and methanol can be found in the supplementary material (Table SI 4). It is also worth noting that while mixture #4 rendered a stable NADES, mixtures containing substantially different amounts of methanol, either in excess or defecit, were not stable: 1:1:1 mixtures crystalized as soon as the vial reached room temperature and 4:1:1 mixtures crystalized within a few hours at room temperature. This simple experiment illustrates the value of the proposed approach that is not only able to identify the compounds required to form a NADES but also the most likely ratios to render stable mixtures.

Moreover, one could imagine that given the appropriate resourses (funding and time), the treshhold could be decreased from the currently 80.1% to render still more useful mixtures. In addition, it is worth mentioning that the formation and physicochemical properties of the NADES formed depend not only on the chemical nature of its components but also on the strength of the intermolecular interactions formed at specific molar ratios. Considering that NADES are formed only when these intermolecular interactions are dominant, one could envision that a further optimization of mixtures #5 and #10 (slightly adjusting molar ratios and/or tailoring the preparation conditions) could lead to the formation of stable mixtures.

### Performance of the optimum classifier on testing dataset

The performance of classifier Alpha was also investigated by means of the test dataset. This dataset is composed of 145 mixtures containing stable and unstable eutectic mixtures reported from the literature. The results are shown in Table 2.

**Table 2:** Comparison of the performance parameters for the Alpha and Gamma classifiers.

Classifier	MCC	Accuracy	F1-score	Loss
Alpha (optimum)	0.42	0.82	0.82	0.56
Gamma (overfitted)	0.42	0.73	0.73	0.70

These parameters were calculated using the same test dataset.

Taking in consideration all the parameters described above, classifier Alpha presented a satisfactory performance evaluating a dataset never seen before by the model. This performance could be improved by increasing the quality as well as the amount of the data present in either the large general chemistry pre-training dataset or the much smaller NADES-specific fine-tuning dataset. The training time would increase in either case, but much more in the case of the large general chemistry dataset—possibly to the point that this strategy would be less attractive even for high end personal computers. Of these two possibilities to further improve the performance of the model, enriching the general-chemistry dataset would be far more costly and in-all-likelihood unnecessary, given the initial success of the model so far. Accordingly, the evidence strongly suggests that growing the NADES-specific dataset used for the fine-tuning process would render a larger impact and enable much more efficient improvement of the algorithm without the need for costly and energy-inefficient computational resources.

As a further note; the predictive power of this model can be improved through the accumulation of more data, and the model itself can be used to optimize the process. Through the process of bench-testing the model’s predictions and thereby increasing the amount of available training data, the model will inevitably become more accurate through subsequent rounds of training. Techniques to optimize this process, known as Active Learning, typically rely on bench testing either the least-confident predictions (i.e. those predictions that lie very close to the chosen 0.5 threshold for stability), on a maximally diverse group of test cases, or a combination of these. The result of this process, if good balance is maintained in the bias of the dataset and care is taken to avoid under- or overfitting, could be the overall accuracy of prediction as well as the confidence in individual predictions rising from around 82–90% or higher.

In summary, the current work was motivated by the need to develop a computationally and energy efficient approach for formulating new natural deep eutectic solvents (NADES). Forming these solvents would be the first step towards their application in the pharmaceutical, agricultural, and food industries. Towards that goal, a transformer-based neural network model was first pre-trained to recognize chemical reaction patterns from SMILES representations (unlabeled general chemical data) and then fine-tuned to recognize the labelled patterns of mixtures known to lead to the formation of either stable or unstable eutectic solvents using binary classification. This strategy, using a comparatively small database (1000 inputs) and a data augmentation strategy, enabled the prediction of multiple new stable eutectic mixtures (n = 337) from a general database of natural compounds. We present a critical assessment of the training process as well as the results of the prediction (components and molar ratios) needed to render NADES with ibuprofen, a molecule that was not present in the original database. Examining the results, the 10 mixtures with the highest predicted likelihood of forming stable NADES were prepared, rendering a success rate of 80%; a figure which strongly validates both the overall accuracy of the model (calculated at 82% on the test dataset) and the model’s confidence that individual mixtures will be stable (a predicted mean of 82.8% for the tested mixtures). While further experiments are needed, it is reasonable to expect that such liquid preparations of ibuprofen and other bioactive compounds could significantly impact the pharmaceutical and nutraceutical industries, as the absorption of many drugs and natural bioactive compounds have been historically hindered by solubility issues. More importantly, this strategy has the potential to provide transformative solutions to the pharmaceutical and nutraceutical industries, where bioactive compounds can become functional components of liquid formulations, rather than simple solutes dispersed in a NADES matrix^91^. We also believe that, with the appropriate databases, the approach could be expanded to predict additional information related to the formation of NADES. That said, this report represents a leap forward towards the efficient development of the newest class of DES: therapeutic DES or THEDES^81,92,93^.

## Methods

### Hardware configuration

All the results presented in this manuscript were generated using the Palmetto cluster, from Clemson University (palmetto.clemson.edu). A NVIDIA Tesla V100 was used as graphical processing unit (GPU) to train and fine-tune the deep learning model. The Palmetto computer node was set to 16 cores (ncpus) and the amount of memory was set to 125 Gb. It is important to state that while access to the cluster was critical to speed up the initial training process, the trained algorithm can be executed in a standard computer.

### Deep learning model

The Hugging Face open-source version of Google Research’s ELECTRA^94^ deep learning transformer was used to train a general chemistry model from scratch and subsequently to fine-tune the model to enable performing downstream tasks such as binary classification. The rational design behind ELECTRA consists of pretraining a discriminator transformer model that predicts tokens either replaced or not from another neural network called the generator. This strategy allows the development of small models that still perform well compared to traditional state-of-the-art natural language processing models such as GPT, BERT-Base, and RoBERTa, given the same dataset. This unique feature also allows the use of relatively small datasets and less computational power to train accurate models. The installation steps and the required packages can be found elsewhere (https://huggingface.co/docs/transformers/installation).

### Chemical databases for AI

#### MIT mixed augmented

The Molecular Transformer MIT Mixed Augmented database^55^ was used to train the general chemistry model from scratch. This database consists of approximately 10^6^ organic reactions, represented using the SMILES^54^ notation. Each line of the source database that contains reactants (*src-train.txt*) is linked to its corresponding products on the target database (*tgt-train.txt*). These two text files were merged into a single raw database (*raw_MIT.txt*) where the reactants are separated from the products by the non-SMILES character “ > ”. The same strategy was used for the test dataset and the resulting file (raw_MIT_test.txt) was used for the proposed general chemistry model.

#### uACL NADES/DES non-augmented

The database developed in-house for this project (referred to as uACL DB) contains approximately 10^3^ previously reported examples of NADES/DES, where the components are represented using the canonical SMILES notation. Those combinations leading to stable mixtures (e.g., synthesized NADES and/or DES in the liquid state that are stable for more than one week at room temperature) were labelled as “1”. On the contrary, combinations of components not leading to liquid mixtures, or those that crystalize soon after the synthesis (non-stable) were labelled as “0”. A fraction (20%) of the raw uACL database was randomly sampled out from the original database to constitute the test dataset. The remaining 80% was then saved in a different file (the training dataset) and used to fine-tune the general chemistry model into a binary classifier, capable of classifying mixtures as stable (1) or non-stable (0). Additionally, both datasets were algorithmically compared to delete any duplicate entries.

#### uACL NADES/DES augmented

In preliminary experiments, we found that the limited size of the database, containing ~ 10^3^ examples of previously-reported (most of them stable) NADES, led to significant overfitting. In this case, the algorithm was able to obtain relatively high scores, even if predicting “stable” for non-stable mixtures. To address this problem, the uACL database was augmented by a script called Mixture Generator Alpha (uACl_mix_gen_alfa.py). The script is responsible for generating mixtures by randomly varying the number of components (from 3 to 5), varying each individual chemical component (among 198 possibilities), as well as the stoichiometric coefficient for each component (from 1 to 10). The mixtures generated by this strategy were labeled as “0” (unstable) and then added to the uACL database according to the number of data augmented (*e.g.* 1, 10, 25, 100, 500). It is also worth mentioning that the number of components was adjusted (from 3 to 5) to increase the likelihood of forming new NADES rather than commonly reported binary DES systems based on choline chloride^40,83^ or ammonium salts^49–52^.

#### Pre-training method

With the recent emergence of transformer-type architectures as a dominant form of Neural Network in the Natural Language Processing space, it has become a standard practice to pre-train these neural nets as Foundation Models of one or more human language(s), using Self-Supervised Learning. State-of-the-art transformer language models are often trained on hundreds of millions or billions of lines of text, at great cost in computer time and energy. This costly pre-training on a general language task instills the model with a broad general “understanding” (*i.e.* statistical characterization) of the language(s), which makes it possible to much more quickly and efficiently fine-tune the model for many potential “downstream” specific tasks such as sentence classification, question answering, etc. In a similar way, the team’s intention here was to use a large general corpus of chemical reaction information in the form of sequences of characters, to pre-train a general chemistry model which could then be fine-tuned on a much smaller dataset for a very specific task. Recent work^95–97^ has demonstrated that such AI approaches can outperform both traditional Force Field and Quantum Mechanical simulations of reaction chemistry for a given amount of computation and reaction complexity. However, unlike the practice of natural language processing, AI Foundation Models for general chemistry are not yet readily available: hence the necessity of the chemistry-specific pre-training effort. The number of hidden layers for the generator as well as discriminator for the ELECTRA deep learning model were 4 and 16, respectively. The vocabulary size was set to 30,000 and the number of training epochs (the number of rounds of training on the full training dataset) to 40. The *train_MIT.txt* file was used as training dataset while *test_MIT_.txt* was used as test dataset. The output model containing all the trained parameters (e.g., discriminator, generator, and vocabulary) was archived in a single directory denominated as model_001.

#### Fine-tuning method

In order to fine-tune the general chemistry model and use it as a binary classifier a custom script was used (binary_model.py), developed following stablished procedures (https://simpletransformers.ai/docs/binary-classification/). The last layer of neurons of the model_001 was fine tuned into a binary classifier by using the train_uACL_non_aug.txt database as training dataset and the test_uACL_non_aug.txt file as test dataset. Additionally, all the augmented test datasets described in item 2.3.3 were used to investigate the performance of those models given the same test dataset (test_uACL_non_aug.txt). Regarding the neural network architecture, the parameters “max_seq_lenght”, ”train_batch_size”, and “learning_rate” were adjusted to 128, 32, and 4E^-5^, respectively.

#### uACL software

**Figure 8:**
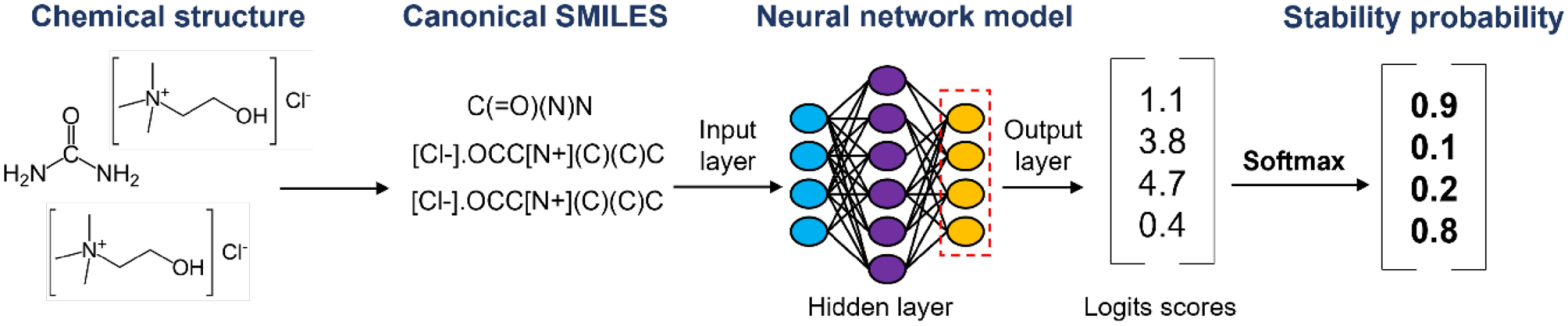
Summary of the proposed strategy for predicting the formation of stable NADES.

To predict the stability of previously-unseen potential DES mixtures, the uACL software was developed. The software is composed by three main modules: Mixture Generator Beta, Classifier, and Compound Finder. As the name suggests, the Mixture Generator is responsible for generating mixtures with a random number of components, random component compounds, and random stoichiometric numbers. Differently from the Mixture Generator Alpha described in item 2.3.3, the Beta version will not assign any label to the combination generated and all the results are saved in a text file (NADES/DES_universe.txt). This text file is then sent to the Classifier, which infers the probability of each mixture to be stable or not. This is accomplished by implementing a SoftMax function^98^ on the raw output of the last layer from the deep neural network model. All the predictions with their respective stability scores are postprocessed in the Compound Finder module. This module allows the user to analyze and predict the eutectic stability of large numbers of mixtures, optionally including a single specified compound (e.g., only mixtures that contain Ibuprofen) in the CSV format. A summary of the proposed strategy is shown in Fig. 8.

#### Chemical reagents

Solid ibuprofen was purchased from Spectrum Chemical Mfg. Corp. (New Brunswick, NJ, USA). Sodium acetate, undecanoic acid, 1,2 butanediol, propionic acid, 1,6 hexanediol, proline, diethanolamine, and ethylene glycol were purchased from Sigma-Aldrich (Burlington, WI, USA). Methanol was purchased from Thermo-Fischer Scientific (Fischer Chemical, NJ, USA). These reagents were of analytical grade (or better) and used as received.

#### NADES/DES preparation

Prior the preparation of NADES/DES mixtures, the individual solid samples were heated at 80 °C for several hours to remove water molecules. NADES and/or DES with molar ratio compositions predicted by the artificial neural network model were prepared by the traditional heating method (80 °C) under magnetic stirring (350 RPM) for 2 h and then allowed to cool down to room temperature.

### Supplementary Information

Supplementary Information.

## Data Availability

The datasets used and/or analysed during the current study available from the corresponding author upon reasonable requests.
