# Supplementary material for: Predicting the formation of NADES using a transformer-based model
Source: Sci Rep. 2024 Feb 22;14:2715. doi: 10.1038/s41598-022-27106-w (PMC10883925; doi:10.1038/s41598-022-27106-w)
Supplement: Supplementary file 1 — Supplementary Information. [file 41598_2022_27106_MOESM1_ESM.docx]

*Supplementary Information for*

**Predicting the Formation of NADES Using a Transformer-Based Model**

Lucas B. Ayres, Federico J. V. Gomez, Fernanda Silva, Jeb R. Linton, and Carlos D. Garcia

| Table SI 1: Confusion matrix for the evaluation dataset at 15 epochs and 100 added synthetic data (n=145) | | | |  | Table SI 2: Confusion matrix for the evaluation dataset at 15 epochs and 500 added synthetic data (n=225) | | | |
| --- | --- | --- | --- | --- | --- | --- | --- | --- |
|  |  | **Actual values** | |  |  |  | **Actual Values** | |
|  |  | **Positive** | **Negative** |  |  |  | **Positive** | **Negative** |
| **Predicted values** | **Positive** | 82 | 24 |  | **Predicted values** | **Positive** | 83 | 31 |
|  | **Negative** | 13 | 26 |  |  | **Negative** | 11 | 99 |

| Table SI 3: Most unstable mixtures predicted by the classifier Alpha | | | | | | |
| --- | --- | --- | --- | --- | --- | --- |
| Component 1 | **Component 2** | **Component 3** | **Component 4** | **Component 5** | **Molar ratio** | **Stability** |
| Mannose | Ibuprofen | Salicylic acid | Levulinic acid | Octadecenoic acid | 4:3:2:3:4 | <0.5 |
| Dimethylurea | Acetic acid | Acetamide | Ibuprofen | Benzoic acid | 3:3:3:2:5 | <0.5 |
| Resorcinol | Glucuronic acid | Ibuprofen | PABA | Aminoacetic Acid | 4:3:2:5:2 | <0.5 |
| Formylphenol | Decaprenoic acid | Lauric Acid | Ibuprofen | - | 3:5:2:1 | <0.5 |
| Propionic acid | Beta Alanine | 3-Chlorobenzoic acid | Ibuprofen | Tryptophan | 4:4:1:3:4 | <0.5 |

| Table SI 4: Chemical properties of the components present in mixture #8. | | | |
| --- | --- | --- | --- |
| component | HYDROGEN BOND DONOR COUNT | HYDROGEN BOND ACCEPTOR COUNT | MELTING POINT (ºC) |
| iBUPROFEN | 1 | **2** | 75.0 - 77.5 |
| Sodium acetate | **0** | **2** | 324.0 |
| methanol | **1** | **1** | -97.6 |
